# Suboccipital double barrel twin meningocoele: Another new theory?

**DOI:** 10.4103/1817-1745.76109

**Published:** 2010

**Authors:** Puneet K. Goyal, Daljit Singh, Hukum Singh, Monica Tandon

**Affiliations:** Department of Neurosurgery, GB Pant Hospital, New Delhi, India

**Keywords:** Double meningomyelocoele, embryogenesis, neural tube defect

## Abstract

Meningomyelocoele (MMC) forms one of the commonest forms of neural tube defect (NTD). It commonly affects lumbosacral area. Double or triple MMC has been reported at various sites of the spine. This supports multiple site closure of neural tube. We report a case of double MMC located at back of head, adjacent to each other like twin MMC. To our best knowledge, such defect has never been reported in the literature and raises query of our current understanding of embryogenesis of NTDs.

## Introduction

Multiple meningomyelocoele (MMC) is a rare entity. There are a few reports in literature where double or triple MMC has been reported by various authors.[1–8] In all these reports, a swelling of one level has been associated with another at a level higher or lower to each other. Moreover, all these reports are on concomitant occurrence of two or more swellings at two different levels of spine.

We came across a rare case where two such swellings were located in suboccipital region, were adjacent to each other, almost symmetrical giving a double barrel appearance. Such observation may help in the future for a better understanding of embryogenesis of neural tube defect (NTD).

## Case Report

**Figure 1:**
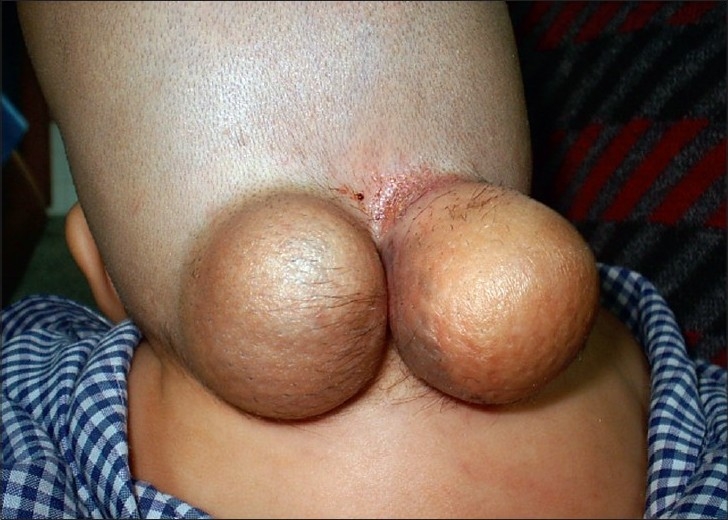
Double barrel meningomyelocele at back of head. Both swellings are almost identical looking

Three-month-old male child presented with two swellings at back of head since birth. The swellings measured 5 × 6 cm and 5.5 × 6.5 cm, and were adjacent to each other without any connection at the base. The swellings were soft, transilluminant and had positive cough reflex. The skin over the swelling was complete and normal. Head circumference was 36 cm, and anterior fontanelle was open. There was no discharge from either of the swellings [Figure 1]. The antenatal ultrasound had suggested a single swelling at neck.

**Figure 2:**
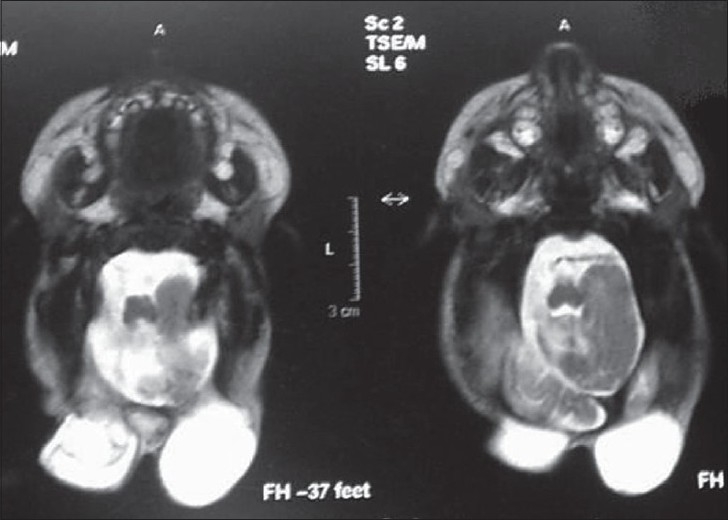
MRI image showing two separate sacs without any neural tissue in them. Note the normal piece of bone in between the two sacs (arrow)

Magnetic resonance imaging (MRI) scan revealed two sacs separated by a small piece of occipital bone, filled with cerebrospinal fluid (CSF) in suboccipital region, with no evidence of any brain tissue in it [Figure 2]. There was no evidence of Arnold–Chiary malformation or hydrocephalus.

**Figure 3:**
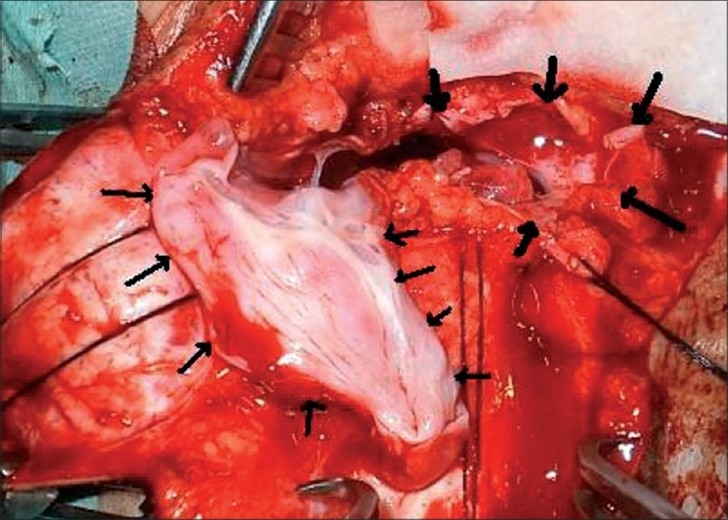
Intraoperative picture of the patient showing two separate sacs (thin and thick arrows)

Patient was operated under general anesthesia in prone position. Transverse skin incision was given. There were two small bony gaps, one each of the swelling in the occipital bone. Operative observations included two separate sacs of CSF, with the left one larger than the right one, and with no communication of two sacs with each other. The small piece of bone in between the two swellings was nibbled out and both sacs were converted into single sac at the base [Figure 3].

The repair was done joining the two sacs as a single opening covering the central communication. The child had a good recovery and had no hydrocephalus at follow-up of 2 years.

## Discussion

It is known that the human embryo passes through 23 stages after conception, each lasting nearly 2–3 days.[9,10] The central nervous system appears during third week as a slipper shaped plate of thickened ectoderm, as the neural plate, at mid dorsal region. Its lateral edges soon elevate to form neural folds. The plate and folds are formed in stage 8 and stage 9 of embryonic development, respectively.[11]

At stage 10, further development results in the neural folds’ elevation, approach and finally fusion in mid line to form neural tube. This process is called as primary neurulation. Fusion begins in cervical region and proceeds in the cephalic and caudal directions until the tube remains open only at small areas at both ends, i.e., anterior and posterior neuropores which close by 25^th^ and 27^th^ days, respectively.[12] The lowest part of spinal cord is formed by secondary neurulation.

The popular theory is that MMC originates from defective closure of caudal neural tube between 26 and 28 days of gestation.[12,13] By this theory, however, there can only be two sites of NTDs – the sites of anterior and posterior neuropores.

However, recently, multiple initiation sites of neural tube closure have been demonstrated in mice and other animals. In humans, Van Allen *et al*.[14] proposed a multisite neural tube closure model in which five closure sites have been shown to be existing in the neural tube of human embryo. The multisite neural tube closure model suggests the existence of five separate closure points, or “zippers,” in normal neural tube in humans. In other words, it indicates the existence of additional neuropores, which are the most frequent site for the defect. These five different “zippers” span the length of the neural tube and function to close it during development. For different levels of defects, the perturbation in the neurulation process occurs in a defined part of the neural tube. These zippers are presumably under the control of one or more genes, mutations in which would cause NTD in the region of that zipper.[8,11] This may explain the embryogenesis of concomitant double meningocoeles.

However, Tomoko Nakatsu *et al*.[15] studied human embryos and found that human neural tube closure sites were different from those of other species. He examined human embryos in which the neural tube was closing grossly and histologically and observed that closure initiates at multiple sites. In addition to the future cervical region that is widely accepted as an initiation site of neural tube closure (site A), the mesencephalic– rhomboncephalon boundary (Site B) was found to be another initiation site. The second closure at site B proceeds bidirectionally and its caudal extension meets the first closure from site A over the rhomboncephalon. The rostral extension of second closure (site B) meets another closure extending from the rostral end of neural groove (site C) over the procencephalon where anterior neuropore closes.

The existing knowledge of zipper and other theories of NTD can explain the findings of more than one MMC at different levels. However, it fails to explain the embryological genesis of two adjacent swellings as in our case. The same can however be explained if there is an existence of neural tube closure in Y-shaped fashion (zipper) at rostral end, i.e., site B of Tomoko Nakatsu *et al*.[15]

It may also suggest that fusion at cephalic level can be in two separate tracts in a Y-shaped manner, the failure of which may result in double barrel defect as in our case. It is a unique case – first in the world which certainly opens rethinking on mechanism of NTDs. The two sacs are adjacent to each other, almost symmetrical in shape and size, and are separated by a part of the occipital bone suggesting the mesoderm involvement in restricting the communication between the two sacs.
